# Nutritional Support with Omega-3 Fatty Acids in Burn Patients: A Systematic Review with Meta-Analysis of Randomized Controlled Trials

**DOI:** 10.3390/nu14142874

**Published:** 2022-07-13

**Authors:** Tippawan Siritientong, Daylia Thet, Maneechat Buangbon, Pawinee Nokehoon, Nattawut Leelakanok, Janthima Methaneethorn, Apichai Angspatt, Jiraroch Meevassana

**Affiliations:** 1 Department of Food and Pharmaceutical Chemistry, Faculty of Pharmaceutical Sciences, Chulalongkorn University, Bangkok 10330, Thailand; daliathet@gmail.com; 2 Center of Excellence in Burn and Wound Care, Chulalongkorn University, Bangkok 10330, Thailand; maneechat@docchula.com (M.B.); noonpawinee@hotmail.com (P.N.); aangspatt@hotmail.com (A.A.); drjiraroch@gmail.com (J.M.); 3 Division of Clinical Pharmacy, Faculty of Pharmaceutical Sciences, Burapha University, Chonburi 20131, Thailand; nattawut.le@go.buu.ac.th; 4 Pharmacokinetic Research Unit, Department of Pharmacy Practice, Faculty of Pharmaceutical Sciences, Naresuan University, Phitsanulok 65000, Thailand; janthima.methaneethorn@gmail.com; 5 Center of Excellence for Environmental Health and Toxicology, Naresuan University, Phitsanulok 65000, Thailand; 6 Division of Plastic and Reconstructive Surgery, Department of Surgery, Faculty of Medicine, Chulalongkorn University, Bangkok 10330, Thailand; 7 Department of Anatomy, Faculty of Medicine, Chulalongkorn University, Bangkok 10330, Thailand

**Keywords:** burn, fish oil, meta-analysis, nutrition, omega-3 fatty acid

## Abstract

Background: In burn patients, the profound effect of nutritional support on improved wound healing and a reduced rate of hospitalization and mortality has been documented. Fish oil as a primary source of omega-3 fatty acids in nutritional support may attenuate the inflammatory response and enhance immune function; however, unclear effects on the improvement of clinical outcomes in burn patients remain. Methods: The systematic literature review was conducted by searching the electronic databases: Cochrane Library, PubMed, ScienceDirect, and Scopus to assess the randomized controlled trials of nutritional support with omega-3 fatty acids compared to control diets in patients that presented with burns from any causes. Results: Seven trials were included in this meta-analysis. We found no significant differences in length of stay (LOS) (*p* = 0.59), mortality (*p* = 0.86), ventilation days (*p* = 0.16), gastrointestinal complications—e.g., constipation and diarrhea (*p* = 0.73)—or infectious complications—e.g., pneumonia and sepsis (*p* = 0.22)—between the omega-3-fatty-acid-receiving group and the control/other diets group. Conclusions: We did not find a benefit of omega-3 support in reducing the various complications, mortality and LOS in burn patients. Further studies are necessary to find the effect of nutritional support with omega-3 fatty acids over low-fat diets in this population.

## 1. Introduction

Burns are a common critical health problem with high mortality, mostly in low- and middle-income countries according to the World Health Organization (2018) [1]. There is a growing recognition of hypermetabolism in burn patients that is accompanied by insulin resistance and profound inflammatory responses [2]. Since the energy expenditure is higher in burn patients than in normal people, nutritional support is a critical process in burn injury based on the areas of burn, risk of malnutrition, or any other comorbid conditions [3]. In recent years, the evidence-based nutritional recommendations to improve clinical outcomes in burn patients have been endorsed by the European Society for Clinical Nutrition and Metabolism (ESPEN) [4].

In particular, infectious complications with inadequate nutrient intakes are accompanied by an increased length of stay (LOS), a high risk of sepsis, or other organ deficits leading to a high mortality rate [5,6,7]. Long-chain omega-3 fatty acids have been recognized for their beneficial role in human health due to their roles as essential fatty acids, together with anti-inflammatory, anti-arrhythmic, immunomodulating, cell growth regulation, and other cardiovascular and cognitive effects [8]. Enteral nutrition with omega-3 fatty acid supplementations is promising for reducing the risks of mortality and morbidity in burn patients by attenuating the inflammatory response and modulating the immune functions after burning. The use of fish oil as a primary source of omega-3 fatty acids, predominantly eicosapentaenoic acid (EPA) and docosahexaenoic acid (DHA), was noted to decrease energy expenditure, the length of intensive care unit stay, ventilation duration, and mortality without causing serious adverse effects in critically ill patients with acute respiratory distress syndrome [9,10]. International guidelines have recommended that early enteral feeding of low-fat diets with or without omega-3 fatty acids after severe burn may improve infectious episodes and delay muscle degradation [4,11]. Additionally, parenteral immunonutrition with omega-3 fatty acids in which the formulation is combined with other adjuvants including antioxidants and amino acids can attenuate severe inflammatory responses in burn patients [3,12]. Basically, it is necessary to decide the nutritional regimens in burn patients based on their characteristics and overall clinical outcomes.

The role of nutritional support in burn care is acknowledged nowadays. To date, there are limited systematic reviews and meta-analyses on the clinical outcomes of omega-3 fatty acid supplementation in the burn population. The pathophysiology of a burn is very complicated, fluctuated, and different from other critically ill patients. Likewise, the benefit of this immunonutrient over low-fat, high-protein diets may not have been elucidated. Therefore, this study aimed to assess the effects of nutritional support containing omega-3 fatty acids on the therapeutic outcomes of burn patients.

## 2. Materials and Methods

### 2.1. Search Strategies

The Preferred Reporting Items for Systematic Reviews and Meta-Analyses (PRISMA) statement was followed for conducting the systematic review. We performed a comprehensive literature search of four electronic databases including Cochrane Library, PubMed, ScienceDirect, and Scopus from their inception through November 2021. The search terms used were omega-3 fatty acid, fish oil, nutrition, burn, burn patient, infection, hospitalization, mortality, and complication. The research question was devised using the PICO framework in the studies of patients with any causes of burn (Participants) receiving fish oil or omega-3 fatty acids through any routes either with or without other immunonutrients (Intervention) compared with placebos or other diets without omega-3 fatty acids (Comparison) on clinical outcomes such as mortality and sepsis (Outcomes) (Supplemental Table S1). The protocol of this systematic review and meta-analysis was not registered.

### 2.2. Inclusion and Exclusion Criteria

All published randomized clinical trials that compared omega-3 fatty acids or fish oil through either nutritional support or supplementations versus placebos, that were conducted in patients of any age group presenting with burns from any causes, and that examined the clinical outcomes, especially the mortality and/or other clinical outcomes, e.g., ventilation days, infections, and other complications, were included in this systematic review and meta-analysis. The combination of omega-3 fatty acids with other immunonutrients was included when the amount of given omega-3 fatty acids was clearly specified. Neither the study country nor the time frame restriction was applied. Only articles published in English were included. The exclusion criteria were: (1) review articles or book chapters, (2) conference abstracts, (3) case series or case reports, and (4) non-human and in vitro studies. Three investigators (D.T., M.B., P.N.) independently performed the literature search and screened the titles and abstracts of the studies. Discrepancies were resolved by the first author (T.S.).

### 2.3. Data Extraction

The retrieved records were exported to the citation manager (EndNote 20.2., Clarivate Analytics, New York, NY, USA). Full-text articles that met the inclusion criteria were independently screened by T.S., D.T., M.B., and P.N. The information extracted from the selected articles was the author name, published year, study setting, study design, study participant characteristics, type and size of the burn, type of nutritional support, nutritional information, and clinical outcomes. Disagreements were resolved by thorough discussions among all authors.

### 2.4. Bias Assessment

The methodological quality of the included studies was independently assessed by two authors (T.S. and D.T.) according to the Cochrane Collaboration’s criteria for randomized trials: random sequence generation, allocation concealment, blinding of participants and personnel, blinding of outcome assessment, incomplete outcome data, selective reporting, and other bias [13]. Any disagreements were solved by consensus among all authors. Following these primary domains, studies were classified as “Low risk (+)”, “Unclear risk (?)”, and “High risk (-)” (Supplemental Figure S1).

### 2.5. Outcome Measurements

The outcomes of this systematic review and meta-analysis were overall mortality, ventilation days, LOS, infectious complications (bacteremia, pneumonia, sepsis, urinary tract infection, and wound infection), and gastrointestinal (GI) complications (constipation and diarrhea) between the omega-3 fatty acids/fish oil groups and control groups. All parameters were determined according to the authors’ definitions in their original articles.

### 2.6. Statistical Analysis

The meta-analysis was conducted using Review Manager (RevMan version 5.4.1: The Nordic Cochrane Center, Copenhagen, Denmark). The pooled unadjusted risk ratio (RR) calculated by the Mantel–Haenszel test with a 95% confidence interval (CI) was estimated for mortality and other complications. The weighted mean difference calculated by the inverse variance method Mantel–Haenszel test with 95% CI was estimated for ventilation days and LOS. The heterogeneity was assessed by I^2^ statistics and was interpreted as high heterogeneity when I^2^ was more than 70% [14]. Including burn patients of all ages and with all kinds of burn injuries may have led to significant heterogeneity between trials. The differences in the results caused by the differences in the model used for data analysis, if any, were also reported. Publication bias was visually evaluated with the funnel plot. Statistical significance was defined as *p* < 0.05.

## 3. Results

**Figure 1:**
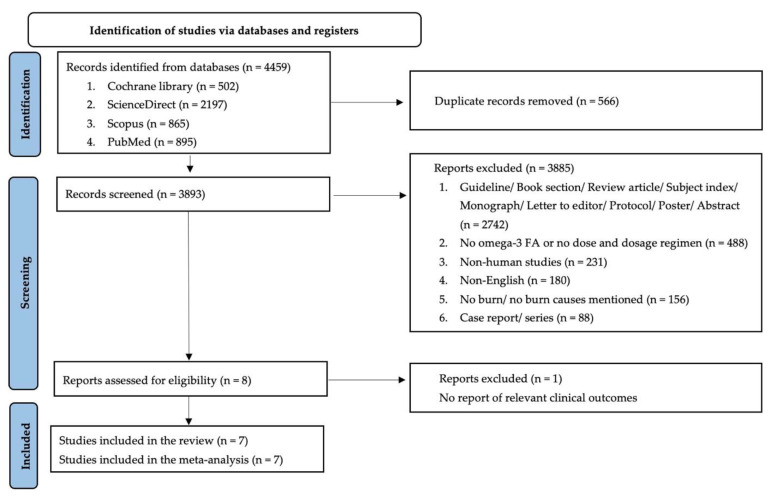
PRISMA diagram for selection and inclusion of the studies.

A total of 4459 articles were identified from the databases. Seven studies were included in this systematic review and meta-analysis (Figure 1). All the included studies were randomized controlled trials of acute burn patients [15,16,17,18,19,20,21]. The characteristics of the included studies are summarized in Table 1. A total of 322 burn patients (143 patients in the omega-3 fatty acids group and 179 in comparators) with an age range of 3 years to 76 years were included in this systematic review. The differences in the amount of energy derived from fat ranged from 15% to 39% of the total energy. Three articles evaluated the sole use of fish oils/omega-3 fatty acids [16,18,21] whereas four articles reported combined immunonutrients [15,17,19,20] in burn patients.

### 3.1. Length of Stay

**Figure 2:**
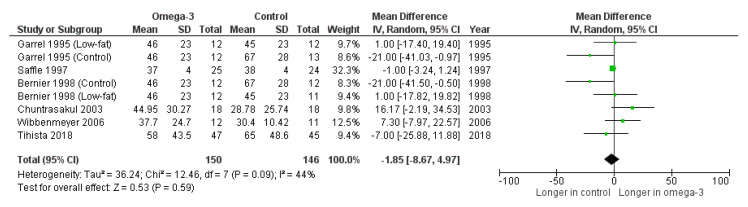
Forest plot of the effects of omega-3-fatty-acid-containing nutritional support on the length of stay [16,17,18,19,20,21].

Garrel et al. [16], Saffle et al. [17], Bernier et al. [18], Chuntrasakul et al. [19], Wibbenmeyer et al. [20] and Tihista et al. [21] reported the mean LOS between the intervention and control groups. The outcome was not significantly different in our pooled analysis of six trials between intervention (126 patients) and control groups (146 patients) (mean difference = −1.85 days, 95%CI: −8.67, 4.97, *p* = 0.59, I^2^ = 44%) (Figure 2).

### 3.2. Mortality

**Figure 3:**
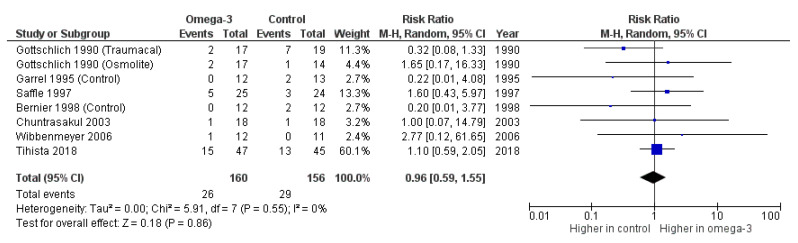
Forest plot of the effect of omega-3-fatty-acid-containing nutritional support on mortality [15,16,17,18,19,20,21].

Mortality outcomes in a total of 299 burn patients were reported by Gottschlich et al. [15], Garrel et al. [16], Saffle et al. [17], Bernier et al. [18], Chuntrasakul et al. [19], Wibbenmeyer et al. [20] and Tihista et al. [21] There were no significant differences in the mortality outcomes of burn patients between the omega-3 fatty acid arm and the control arm (unadjusted RR = 0.96, 95% CI: 0.59, 1.55, *p* = 0.86, I^2^ = 0%) (Figure 3).

### 3.3. Ventilation Days

**Figure 4:**
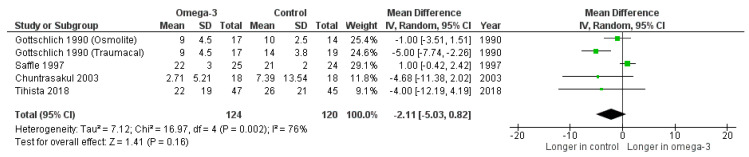
Forest plot of the effect of omega-3-fatty-acid-containing nutritional support on ventilation day [15,17,19,21].

Four trials (Gottschlich et al. [15], Saffle et al. [17], Chuntrasakul et al. [19], and Tihista et al. [21]) reported the number of ventilation days in burn patients receiving omega-3-containing formula and control diets. We found no clinical significance in our pooled data of 227 patients (mean difference = −2.11 days, 95% CI: −5.03, 0.82, *p* = 0.16). However, the heterogeneity was significant (I^2^ = 76%, *p* = 0.002), which is shown in Figure 4.

### 3.4. Gastrointestinal Complications; Constipation and Diarrhea

**Figure 5:**
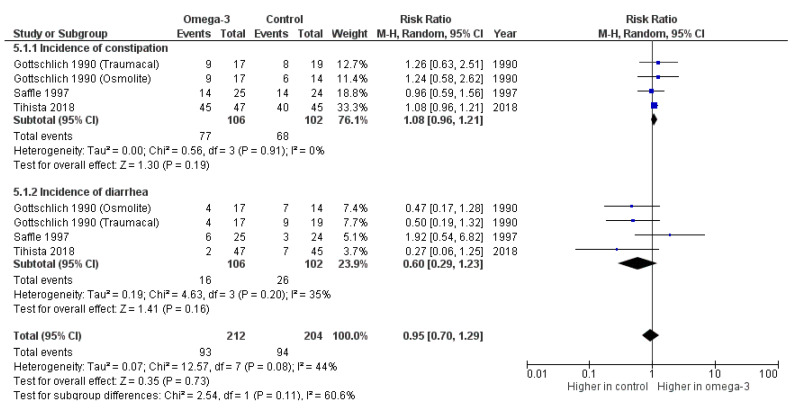
Forest plot of the incidence of gastrointestinal complications following omega-3-fatty-acid-containing nutritional support [15,17,21].

Some GI complications, e.g., constipation and diarrhea from 191 burn patients were documented in three randomized trials [15,17,21]. The occurrence of both constipation and diarrhea was not clinically different between the study group and the control group as shown in Figure 5 (unadjusted RR = 1.08, 95%CI: 0.96, 1.21, *p* = 0.19, I^2^ = 0% and unadjusted RR = 0.60, 95%CI: 0.29, 1.23, *p* = 0.16, I^2^ = 35%, respectively).

### 3.5. Infectious Complications; Bacteremia, Pneumonia, Sepsis, Urinary Tract Infection, and Wound Infection

**Figure 6:**
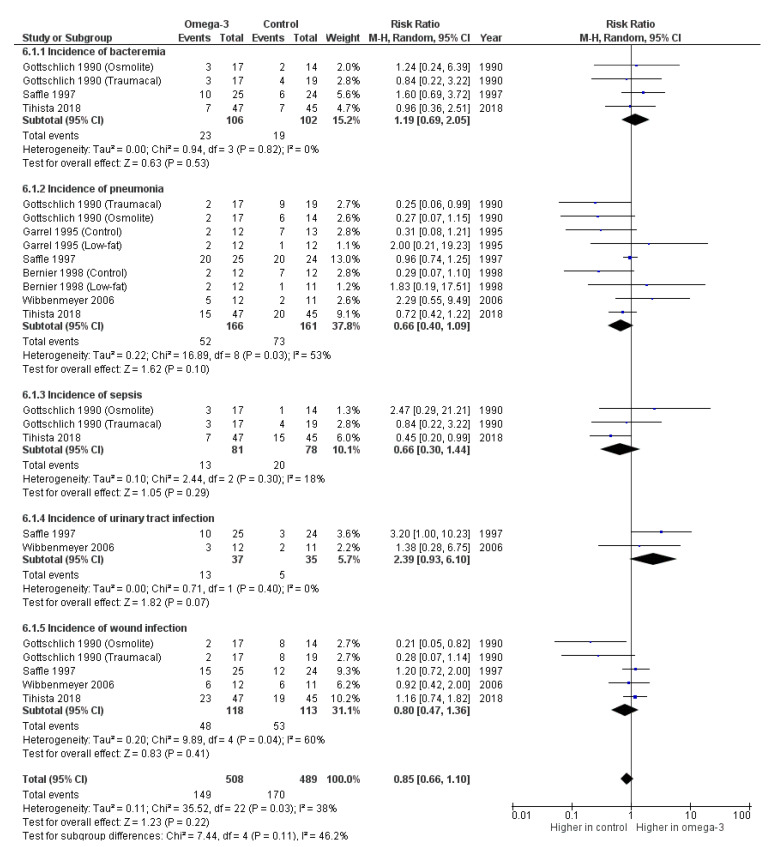
Forest plot of the incidence of infectious episodes following omega-3-fatty-acid-containing nutritional support [15,16,17,18,20,21].

As shown in Figure 6, the overall pooled data on the incidence of bacteremia (191 patients), pneumonia (286 patients), sepsis (142 patients), urinary tract infection (72 patients), and wound infection (214 patients) from six prospective trials [15,16,17,18,20,21] depicted no significant differences between the omega-3-fatty-acid-containing formula and other control diets with the overall unadjusted RR of 0.85 (95% CI: 0.66, 1.10, *p* = 0.22, I^2^ = 38%).

### 3.6. Risk of Bias

**Figure 7:**
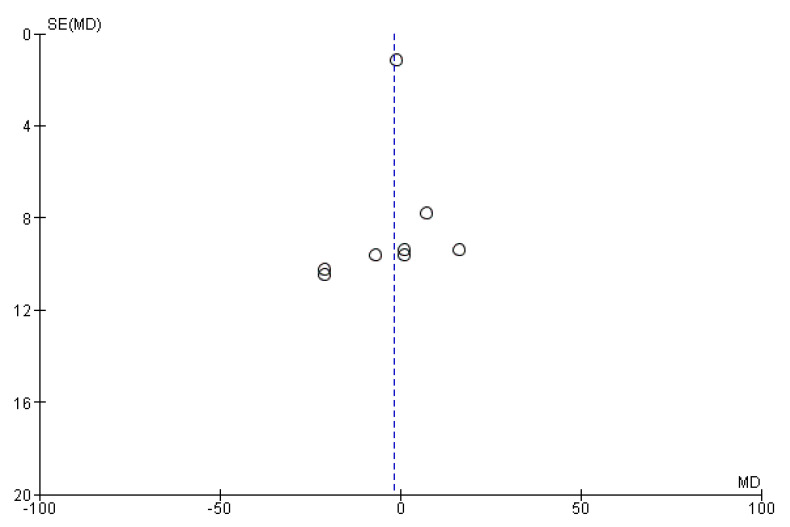
Funnel plot of length of stay (MD, mean difference; SE, standard error).

There were low risks of bias in most criteria (Supplemental Figure S1) despite high detection bias due to the lack of blinding of outcome assessments. Some studies revealed unclear risks of bias in allocation concealment. The visual inspection of the funnel plot showed no potential publication bias in our systematic review (Figure 7).

## 4. Discussion

The ESPEN guidelines pointed out the favorable effect of low-fat (15% of energy requirement), adequate-protein nutritional support on the length of hospital stay, and risk of infection in burn patients; however, the benefit of omega-3 fatty acids over other types of fat remains unclear [4]. This systematic review and meta-analysis did not give evidence for the superior benefits of the nutritional support containing omega-3 fatty acids over other diets regarding LOS, mortality, ventilation days, GI complications, or infections in burn patients.

In the context of burn injury, particularly, elevated immune response and hypermetabolism would aggravate the burn severity and mortality risk [22]. In the human body, omega-3 fatty acids prevent the overreactions of arachidonic acid cascade into pro-inflammatory eicosanoids (prostaglandins, thromboxanes, leukotrienes). As a result, the less inflammatory pathways or the resolution of inflammation could appear after omega-3 fatty acid provision. Regardless of dosage, the overall effect of fish oil parenteral supplementation resulted in decreased mortality in patients with critical illnesses, demonstrating a reduction in the Simplified Acute Physiology Score by 7% (from 18.9% to 11.9%, *p* < 0.001) [23]. This potential advantage of omega-3 supplementation in reducing mortality was supported by the study in ICU patients [24]. Additionally, the ventilation requirements and LOS were also lower in critically ill patients receiving fish-oil-containing parenteral nutrition [23], especially those presenting with acute respiratory distress syndrome [10]. The result agreed with the meta-analysis of Lu et al. [25], which found shorter ventilation days in patients with sepsis receiving omega-3 supplementation. In addition, it was recently found that both the standard administration of fish oil alone and fish-oil-containing parenteral nutrition had a clinical benefit on overall mortality, 28-day mortality, morbidity, length of ICU stay, and infectious complications in critically ill patients [26]. It is also interesting to note that the presence of mechanical ventilation could predict the risk of mortality in burn patients [27]. In addition to the burn wound, inhalation injury is considered an important factor in burn severity and predicts mortality. It was reported that burn patients with inhalation injury had a higher risk of mortality than those without inhalation injury (31% vs 4.3%) [28].

The study by Garrel et al. [16] suggested that omega-3- or fish-oil-containing diets did not have any additional benefit in reducing LOS due to the similar outcomes between fish oil and low-fat diets. Contrarily, the comparative outcomes in different populations receiving omega-3-containing nutritional support were noted. In patients with GI malignancies, postoperative enteral nutrition with omega-3 fatty acids and supplemental arginine improved clinical outcomes by significantly reducing hospital LOS [29]. The combined immunonutrition may provide advantages in specific conditions of traumatic or critically ill patients, but they were not obviously noticed in burn patients with regard to mortality, LOS, and infectious complications [30].

Generally, levels of proinflammatory cytokines are elevated in many burn patients [31]. It has been elaborated that infections such as pneumonia are associated with high levels of interleukin-6 in thermally burn patients, regardless of the type of diet, while there were inconsistent elevations of tumor necrosis factors [18,32]. Gottschlich et al. [15] reported pneumonia as one of the primary causes of death in their burn population, which supported the probability of complication-associated death. However, the incidence was quite lower in a modular diet enriched with the omega-3 fatty acid group. Since omega-3 fatty acids have potential anti-inflammatory actions by reducing immune responses, their benefits of immunomodulation have been explored in previous studies [33,34,35]. Considering the effects of omega-3 fatty acids on infection rates, it was shown that patients receiving omega-3 parenteral nutrition had a low risk of nosocomial infections [36]. In a previous study, clinical benefits such as lower rates of sepsis and GI complications due to a well-tolerated omega-3-fatty-acid-containing diet were well noted [21]. The rates of severe sepsis and septic shock were two times lower in burn patients receiving omega-3 polyunsaturated fatty acids than in the control group [21]. The benefit was demonstrated in other populations such as patients with GI malignancies [29]. The incidence of infections and wound complications were significantly lower in the omega-3-supplemented group than in the control group.

Given the different compositions of fatty acids in nutritional support formulations/diets, their administration in a particular disease condition may vary clinical outcomes. One study included in this systematic review stated omega-6/omega-3 ratios in thermal burn patients (1.5:1 and 3.6:1 in fish oil with the arginine diet group and control group, respectively) [20]. However, the clinical changes between the groups were not significant in their study. A recent study documented the lower incidence of metabolic syndrome and cardiovascular risks in obese people receiving a diet with a low omega-6/omega-3 ratio (<4) compared to those receiving a regular diet [37]. Of note, consuming fatty acids with an omega-6/omega-3 ratio of 6–10 was observed to reduce the risk of overall mortality in the Chinese population [38].

The benefits of omega-3 fatty acids were well noted. However, there were potential adverse effects of this nutrient upon administration by either oral or enteral routes. Typically, omega-3 fatty acid at a high concentration leads to untoward effects including cell damage by autoxidation and hematological disturbances such as platelet aggregation [39]. Recently, an unclear benefit of omega-3 fatty acids was documented in specific groups of patients. The oral supplementation with omega-3 fatty acids provided more harm than benefit in patients with cardiovascular disease: in some cases, an increased risk of atrial fibrillation [40].

Our study, however, is not without limitations. The quality, compositions, or formula of the diets, the omega-3 contents, and the addition of immunomodulators such as arginine were varied in our included studies which probably had an impact on between-group outcomes. Furthermore, the severity of burns such as inhalation injury might differ among patients. The number of randomized trials and the populations involved in this meta-analysis was limited; thus, a subgroup analysis by stratifying the burn size, for instance, was not performed. Several included trials revealed high risks of detection bias in addition to heterogeneity among the population.

Our findings were in agreement with the previous meta-analysis of adult patients with critical illnesses [41]. There were no improvements in mortality, infectious conditions, or LOS following parenteral supplementation containing omega-3 fatty acids. Likewise, enteral nutrition with omega-3 fatty acids did not improve all-cause 28-day mortality, ventilation days, or ICU-free days in populations with acute respiratory distress syndrome [42]. Similarly, the recent meta-analysis evaluating the benefits of omega-3 fatty acids in patients with acute respiratory distress syndrome observed unchanged LOS, mortality, and infection complications [43]. The association between complications and omega-3 fatty acid nutritional support diets is still unclear. It is necessary to prove the role of omega-3-enriched diets to ameliorate the clinical status of many critically ill populations, including burn patients.

## 5. Conclusions

In conclusion, the findings of this systematic review and meta-analysis do not support the superiority of omega-3 fatty acid nutritional support over low-fat, adequate-protein diets. Further research is highly recommended to investigate the association between clinical outcomes and nutritional support with omega-3 fatty acids.

## Figures and Tables

**Table 1:** Description of included studies.

No.	Author, Year of Publication	Study Location	Study Participants	Omega-3 FA/fish Oils Formula Characteristics	Intervention Period	Outcomes of the Intervention	
						Mortality	Others
1	Gottschlich et al., 1990 [15]	USA	50 acute thermal burn patients (10–89% BSA), 0–79% full-thickness injuries - 17 patients in the intervention group, aged 4–76 years) - 33 patients in the control group, aged 3–71 years	Modular tube feeding containing protein (87% whey, 9% arginine, 2% cysteine, 2% histidine), carbohydrate (maltodextrin), fat (50% fish oil, 50% safflower oil): 5 g omega-3 FA in 1029.80 g of diet	- 5 weeks - Enteral nutrition given within 48 h of admission	- The overall mortality was 20%, while 70% of deaths occurred in the group supported with a large dose of fat and linoleic acid.	- Significantly decreased wound infection (*p* < 0.03) - Reduced length of hospital stay (*p* < 0.02), - Marginal effect on incidence of pneumonia (*p* < 0.06) and total number of infectious complications (*p* < 0.07) - No effect on the incidence of clinical sepsis - Low incidence of diarrhea, improved glucose tolerance, low serum triglycerides, and improved maintenance of muscle mass
2	Garrel et al., 1995 [16]	Canada	37 acute thermal burn patients (>20% of BSA) - 12 patients in the intervention group, aged 16–52 years - 25 patients in control or low-fat diet, aged 17–63 years	Low-fat formula 15% fat (50% fish oil, 40% soybean oil, 10% MCT oil); 60% carbohydrate, 25% protein	- 25 days - Enteral and parenteral nutrition given within 24 h after injury	- 2 deaths from the control group	- Low-fat nutritional supplements reduced infection rate, healing time, and length of stay. - Fish-oil-containing nutritional support was not likely to change clinical outcomes compared to a low-fat diet without fish oil.
3	Saffle et al., 1997 [17]	USA	49 acute burn patients (2.5–82.5% BSA) - 25 patients in the intervention group, aged 6–68 years - 24 patients in the control group, aged 6–85 years	The enteral formula containing 22% protein (14 g arginine, and other amino acids), 53% carbohydrate, 25% fat (6% omega-3 FFAs; 1.3 g/L EPA, 0.45 g/L DHA) with 1.2 RNA and 10 g dietary fiber	- 2 weeks - Enteral nutrition within 48 h of injury	- No significant difference in mortality rate between the intervention and control group.	- No significant clinical changes in ventilation days and length of hospital stay between the intervention and control group.
4	Bernier et al., 1998 [18]	Canada	35 thermal burn patients (>20% of BSA) - 12 patients in the intervention group, aged 32.5–37.9 years - 23 patients in control or low-fat diet, aged 32.7–44.5 years	Low-fat formula 15% fat (50% fish oil, 40% soybean oil, 10% MCT oil); 60% carbohydrate, 25% protein	- 28 days - Enteral and parenteral nutrition given within 24 h after admission	- 2 deaths from the control group	- Significant fast recovery and low incidence of pneumonia in patients receiving a low-fat diet with or without fish oil compared to the control group.
5	Chuntrasakul et al., 2003 [19]	Thailand	36 patients (16 trauma and 20 burn); (30–60% BSA) - 18 patients in the intervention group, aged 16.56–41.1 years - 18 patients in the control group, aged 17.65–44.13 years	Enteral formula containing 62.5 g/L protein (70% casein, 20% arginine, 10% glutamine), 125 g/L carbohydrate, 28 g/L fat (20% fish oil, 30% corn oil, 50% MCT oil)	- 11 days - Enteral nutrition given on 2nd day after injury	- One patient in each group died.	- Fish-oil-containing enteral feeding decreased ICU days and wean-off respirator days.
6	Wibbenmeyer et al., 2006 [20]	USA	23 thermal burn patients (>20% BSA) - 12 patients in the intervention group, aged 26.1–58.9 years - 11 patients in the control group, aged 25.7–63.5 years	Enteral formula containing 39% fat (fish oil, soy oil, medium-chain triglyceride; 1.7 g/L EPA, 1.2 g/L DHA), 36% carbohydrate (maltodextrin, corn starch), 25% protein (casein, L-arginine)	- 4 weeks - Enteral nutrition given within 48 h of admission	- 2 deaths in the intervention group, of which 1 event occurred within 24 h of enrollment.	- Patients receiving fish oil and arginine formula had a slightly faster healing time than those receiving a standard diet. However, patients from the intervention group were more likely to get infections and complications. - No difference in length of stay between the two diet groups.
7	Tihista et al., 2018 [21]	Uruguay	Burn patients (>15% BSA) - 47 patients in the intervention group, aged 22.5–54.9 years - 45 patients in the control group, aged 25–58.2 years	Enteral formula containing 18% fat (50% fish oil, 50% sunflower oil), 62% carbohydrate (maltodextrin), 20% protein (casein)	- 2 weeks - Enteral nutrition given within 24 h of admission	- No significant difference between groups.	- A low-fat diet with omega-3 FA showed a significantly lower incidence of severe sepsis, septic shock, and non-infectious complications compared to a standard low-fat diet. - 4 days shorter ventilation days in the intervention group. - No significant length of stay between groups.

BSA, body surface area; DHA, docosahexaenoic acid; EPA, eicosapentaenoic acid; FA, fatty acid; MCT, medium-chain triglyceride.

## Data Availability

The data presented in this study are available on request from the corresponding author.

## Supplementary Materials

The following supporting information can be downloaded at: https://www.mdpi.com/article/10.3390/nu14142874/s1, Figure S1: Risk of bias assessment; Table S1: Search strategy.
