# Supplementary material for: Nutritional Support with Omega-3 Fatty Acids in Burn Patients: A Systematic Review with Meta-Analysis of Randomized Controlled Trials
Source: Nutrients. 2022 Jul 13;14(14):2874. doi: 10.3390/nu14142874 (PMC9320673; doi:10.3390/nu14142874)
Supplement: Supplementary file 1 [file nutrients-14-02874-s001.zip › Omega3-Burn SRMA Supplementary material 1_220629.pdf]

Supplemental Table S1. Search strategy

Database	Search terms
Cochrane Library	("fatty acids, omega 3" OR "omega 3 fatty acid" OR "omega-3 fatty acid" OR "polyunsaturated fatty acid" OR "omega-3 oil" OR "omega 3 oil" OR "ω-3 fatty acid" OR "n-3 fatty acid" OR "docosahexaenoic acid" OR "eicosapentaenoic acid" OR "fish oil" OR nutrition OR "enteral nutrition" OR "parenteral nutrition" OR supplement) AND (burns OR burn OR "thermal burn" OR "chemical burn" OR "electrical burn" OR "radiation burn" OR "inhalation burn" OR "burn patient") AND (mortality OR death OR "length of stay" OR hospitalization OR infection OR sepsis OR "septic shock" OR morbidity OR complication OR "clinical outcome")
PubMed	("fatty acids, omega 3"[Mesh] OR "omega 3 fatty acid"[tw] OR "omega-3 fatty acid"[tw] OR "polyunsaturated fatty acid"[tw] OR "omega-3 oil"[tw] OR "omega 3 oil"[tw] OR "ω-3 fatty acid"[tw] OR "n-3 fatty acid"[tw] OR "docosahexaenoic acid"[tw] OR "eicosapentaenoic acid"[tw] OR "fish oil"[tw] OR nutrition OR "enteral nutrition" OR "parenteral nutrition" OR supplement) AND (burns[Mesh] OR burn[tw] OR "thermal burn"[tw] OR "chemical burn"[tw] OR "electrical burn"[tw] OR "radiation burn" [tw] OR "inhalation burn"[tw] OR "burn patient"[tw]) AND (mortality[tw] OR death[tw] OR "length of stay"[tw] OR hospitalization[tw] OR infection[tw] OR sepsis[tw] OR "septic shock"[tw] OR morbidity[tw] OR complication[tw] OR "clinical outcome"[tw])
ScienceDirect	(((“omega 3 fatty acid” OR "fish oil" OR "omega 3 supplement") AND (burn OR "burn patient") AND ("length of stay" OR infection OR mortality)))
Scopus	(TITLE-ABS-KEY (("omega-3 fatty acid" OR "omega 3 fatty acid" OR "omega-3 oil" OR "omega 3 oil" OR "ω-3 fatty acid" OR "n-3 fatty acid" OR "docosahexaenoic acid" OR "eicosapentaenoic acid" OR "fish oil" OR "omega 3 supplement" OR

	"enteral nutrition" OR "parenteral nutrition")) AND TITLE-ABS-KEY ((burn OR "thermal burn" OR "chemical burn" OR "electrical burn" OR "radiation burn" OR "inhalation burn" OR "burn patient")) AND TITLE-ABS-KEY ((mortality OR "length of stay" OR hospitalization OR infection OR sepsis OR morbidity OR complication OR "clinical outcome")))
