# Supplementary material for: Nutritional Support with Omega-3 Fatty Acids in Burn Patients: A Systematic Review with Meta-Analysis of Randomized Controlled Trials
Source: Nutrients. 2022 Jul 13;14(14):2874. doi: 10.3390/nu14142874 (PMC9320673; doi:10.3390/nu14142874)
Supplement: Supplementary file 1 [file nutrients-14-02874-s001.zip › Omega3-Burn SRMA Supplementary material 2_220629.pdf]

	Random sequence generation (selection bias)	Allocation concealment (selection bias)	Blinding of participants and personnel (performance bias)	Blinding of outcome assessment (detection bias)	Incomplete outcome data (attrition bias)	Selective reporting (reporting bias)	Other bias
Bernier 1998		-	+	-	+	+	+
Chuntrasakul 2003		-	-	-	+	+	+
Garrel 1995	+		+	-	-	+	+
Gottschlich 1990	+		+	-	+	+	
Saffle 1997	-	-	+	-	+	+	+
Tihista 2018	+		+	-	-	+	
Wibbenmeyer 2006	+		+	-	-	+	

+ Low risk of bias
- High risk of bias

Supplemental Figure S1. Risk of bias assessment
